# Prognosis in Coronary Thrombosis

**Published:** 1932

**Authors:** Carey F. Coombs

**Affiliations:** Physician, and in charge of the University Centre of Cardiac Research, General Hospital, Bristol


### PROGNOSIS IN CORONARY THROMBOSIS.

BY

CAREY F. COOMBS, M.D., F.R.C.P.,

*Physician, and in charge of the University Centre of Cardiac Research, General Hospital, Bristol.*

---

NOT so many years ago, if a candidate in one of the higher examinations had been questioned as to the prognosis of coronary thrombosis, he might have answered with impunity that there is none. The past decade has, however, brought to light the fact that many people do survive these catastrophes. Various series of cases have been reported which bear this out, and to these I add notes drawn from 144 patients seen by myself. Examples of sudden death without benefit of medicine, discovered post-mortem to be due to coronary thrombosis, have been excluded from this series; for the object of these remarks is to help the doctor who, standing by the bedside of someone struck down by cardiac infarction, has to furnish to himself and others some sort of estimate of the patient's chances of recovery.

Now, two broad facts emerge from this study. The first is that 49 out of 144, or 1 out of 3, died in or shortly after the attack. Secondly, of the 83 survivors whose attacks occurred over a year ago 51 are still living. In other words, about one-third of those who survive the attack die within the year. It is only fair to add that with increasing experience one is more and more ready to make a diagnosis of coronary thrombosis in relatively mild cases. The present series included none except those in which the diagnosis would command general agreement. If debatable cases were added, these would dilute the fatalities and furnish a better picture of the prospects.

These general data can also be used as tests of the influence of several ætiological factors and clinical features. In all the tables that follow (except the last) the first column refers to the total number of cases reviewed. The second column expresses in percentages the proportion of those who survived the attack itself. The third column refers to the percentage of those who, after living through the attack, had also survived through the subsequent year. The ages of the whole series may conveniently be considered in three groups; those who were under sixty, those who were seventy or over, and those whose ages lie between, in the seventh decade. The following table expresses the influence of age on the prognosis:—

| Age. | Total Number. | Non-fatal attacks. | Non-fatal and surviving a year or more. |
|---|---|---|---|
| Under 60 .. .. | 48 | 66·6% | 51·8% |
| 60–69 .. .. .. | 66 | 75·7% | 71·1% |
| 70 or over .. .. | 30 | 43·3% | 70·0% |

The influence of sex may be tested in the same way :—

| | Total Number. | Non-fatal attacks. | Non-fatal and surviving a year or more. |
|---|---|---|---|
| Males .. .. .. | 106 | 68·8% | 58·0% |
| Females .. .. | 38 | 57·9% | 75·0% |

A family history of liability to what may be broadly called coronary disease is too vague a factor to estimate in figures, yet I believe it is an unfavourable feature. I have seen examples of fatalities in people with this kind of history which seem to me proportionally more numerous than in those who have no such history; but I must admit that I can produce no reliable figures in support of this impression.

The state of the heart before the attack may be supposed to influence the prognosis, but it is difficult, in a majority of cases, to get much precise information about this. However, it is practically always possible to learn whether the patient has or has not been liable to cardiac pain on effort. The present series may from this point of view be divided into a group of 64 who gave a history of this kind, and 80 who did not. In tabular form the prognosis of these two groups may be compared thus :—

| | Total Number. | Non-fatal attacks. | Non-fatal and surviving a year or more. |
|---|---|---|---|
| Attack preceded by pain on effort .. | 64 | 65·6% | 57·1% |
| Not preceded by pain on effort.. | 80 | 66·2% | 64·5% |

All that can be drawn from this series as to the influence of background on prognosis, therefore, is the somewhat obvious fact that patients of seventy and over stand up to this calamity less successfully than do patients who are not so old.

The prognosis, perhaps, depends more on the severity of the attack than on the background. But how is that to be measured ? There are two kinds of evidence, those which arise immediately from the anatomical change in the heart, and those which express the same thing in terms of function—data derived from the state of the peripheral circulation. Now, after examining all the evidence directly proving a structural change in the wall of the heart which was at my disposal, I found that it was not possible to use any simple clinical criterion of gross cardiac damage save one—the presence or absence of a pericardial rub. Here again, therefore, I divided my cases into two groups, those in which friction was heard and those in which it was not. The prognosis in these two groups I express again in tabular form.

| Pericardial friction. | Non-fatal attacks. | Non-fatal and surviving a year or more. |
|---|---|---|
| Heard in 42 . . . . | 57·1% | 70·0% |
| Not heard in 102 . . | 75·4% | 58·7% |

The cases in which a pericardial rub is heard are, therefore, rather worse off so far as the immediate prospect is concerned than those in which it is not heard. Again, the outlook is, perhaps, a little less hopeful when the pain is abdominal than when it is thoracic, as the following table shows :—

| | Total Number. | Non-fatal attacks. | Non-fatal and surviving a year or more. |
|---|---|---|---|
| Pain abdominal . . | 28 | 57·1% | 64·2% |
| Pain thoracic . . | 116 | 68·1% | 60·8% |

The influence on the prognosis of the electrocardiographic picture is not easy to state in terms of figures; in fact, it would be impossible to do so, as far as my own experience has gone, because it has not always been possible to collect the necessary data. So often the patient is too ill for early electrocardiography. Persistent ventricular tachycardia is generally admitted to involve a bad prognosis. Probably it is true here, as in cardiac disease in general, that the most serious changes are those which prove the greatest damage to the ventricular walls. Equally it is true that the course of the case towards recovery can be estimated more accurately if electrocardiograms are taken at intervals.

The immediate danger in cardiac infarction is that the heart will not be able to fill the peripheral circulation. It is accordingly to the signs of that failure that we should turn for a measure of the prospect of recovery. It is this that makes extreme pallor so ominous a symptom. The rate of the pulse is not a good guide. I have known the heart beat at a normal rate in a fatal case up to the moment at which breathing ceased to be rhythmic, a few minutes before the cardiac sounds disappeared. On the other hand, measurement of the blood-pressure nearly always gives reliable evidence of the extent to which the efficiency of the peripheral circulation is impaired. The most significant feature of the blood-pressure in coronary thrombosis is the fall in the systolic tension. It is not always possible to be sure that this has taken place, for in many instances the pressure before the attack was unknown; but what is, after all, the most direct measure of the efficiency with which the damaged heart is filling the vessels is the pulse-pressure. My custom (based on a long series of actual data) is to regard the pulse-pressure as normal if it is about 40 per cent. of the systolic pressure. In coronary thrombosis, however, and other forms of severe ventricular failure, it may fall as low as 10 per cent. The following table shows that a fall to 25 per cent. or below is a bad sign:—

| | Pulse pressure/Systolic pressure =25 per cent. or less; bearing on prognosis. | |
|---|---|---|
| | Died in attack. | Died in year following attack. |
| Whole series (144).. .. | 34·0% | 38·5% |
| P.P. low (27) .. .. .. | 70·4% | 100·0% |

Unfortunately, it is not possible to claim the converse, that patients with a wide pulse-pressure are sure to recover; yet if I were compelled to rely for prognosis on one sign alone it would be the pulse-pressure that I should choose. Curiously enough, an alternating pulse is seldom found except when the immediate attack has been survived and secondary ventricular failure has set in. I say "curiously" because narrowed pulse-pressure and an alternating pulse are so often associated in the progressive ventricular defeat of the hyperpietic. There is no time to discuss in detail the course followed by signs and symptoms in those who survive the initial onslaught. The more evidence there is of damage to ventricular muscle and reduction of ventricular output the worse the prognosis. Persistence of fever and of leucocytosis are bad signs. Returning vigour of sounds and beat and rise in the pulse-pressure are favourable. Of complications, glycosuria and embolic accidents make the outlook graver still.

The ultimate outcome in those cases surviving both the attack and the subsequent year cannot yet be summarized statistically, because the period during which they have been observed is not long enough. It is only fair to claim, however, that in many of these a recovery that is apparently complete has taken place. That is to say, the patients have been enabled to resume and carry on with their work provided it is not too arduous, and to survive the attack by at least a decade. This is in accordance with the experience of those who have followed up similar series. The same lesson is taught when post-mortem evidence brings to light the cicatricial remains of cardiac infarction, which must have occurred many years before death.

Finally, there is no doubt that with an increasing accuracy of early diagnosis the prognosis is improving. Early diagnosis leads to thorough treatment, based on an adequate period of rest, and in this way an appreciable number of lives are saved.

### SUMMARY.

Of a series of 144 clinically diagnosed cases of coronary thrombosis approximately one-third died in or shortly after the initial attack.

Of those who survived the attack about one-third died during the ensuing year.

The severity of the attack appears to have more prognostic import than the state of the patient before the attack.

The degree to which the pulse pressure falls is a good index of the severity of the attack.

EDITOR'S NOTE.

A sad interest attaches to this paper, the proofs of which were returned by Dr. Carey Coombs on the day before his death with a letter saying that he was sending " an alternative paragraph to the one already in my paper. I think it is only fair to others in the same boat to dwell a little more than I have done on the bright side." The paragraph in question has been added in brackets on page 283.

